# Postoperative outcomes following anatomical lung resections in relation to intraoperative ventilation practices: a registry-based multicenter study

**DOI:** 10.3389/fsurg.2026.1740340

**Published:** 2026-07-15

**Authors:** Timon Marvin Schnabel, Mark Schieren, Jerome Defosse, Mark Ulrich Gerbershagen

**Affiliations:** 1 Department of Anaesthesiology, University Witten/Herdecke, Cologne-Holweide Hospital, Cologne, Germany; 2 Department of Anaesthesiology and Intensive Care Medicine, University Witten/Herdecke, Cologne-Merheim Hospital, Cologne, Germany

**Keywords:** anatomical lung resection, intraoperative ventilation, lung-protective ventilation, mechanical ventilation, one-lung ventilation, pulmonary complications

## Abstract

**Background:**

Patients undergoing anatomical resection during thoracic surgery are exposed to an elevated risk of postoperative complications, particularly when one lung ventilation (OLV) is applied. The objective of this study is to evaluate the association between intraoperative ventilation parameters during OLV in anatomical resections and outcomes in this cohort.

**Methods:**

A retrospective multicenter cohort study was conducted using data from the German Thoracic Registry. Between 2015 and 2021 1128 patients underwent anatomical resection. Intraoperative parameters analyzed included driving pressure (DP), peak airway pressure (Pmax), positive end-expiratory pressure (PEEP), and tidal volume per predicted body weight (TV/PBW). The primary outcomes of the study were postoperative complications, respiratory complications, and in-hospital mortality.

**Results:**

The analysis included 1128 patients undergoing anatomical lung resections, with a postoperative complication rate of 28.7% and respiratory complications in 16.3%. Elevated DP >20 mbar (*p* = .008), Pmax >25 mbar (*p* = .003), and TV >5 mL/kg PBW (*p* = .013) were significantly associated with increased complication rates. Respiratory complications were significantly associated with DP >20 mbar (*p* = .006), Pmax >25 mbar (*p* = .001), and PEEP >8 mbar (*p* = .017). In-hospital mortality was 1.2% (*n* = 14). Given the low number of events, mortality analyses were considered exploratory; unadjusted analyses showed higher mortality with prolonged OLV and longer surgery duration (both *p* < .001).

**Conclusion:**

In this registry-based cohort, univariable threshold analyses indicated higher postoperative and respiratory complication rates when intraoperative airway pressures and tidal volumes exceeded commonly used protective ranges. However, these findings should be interpreted as observational and hypothesis-generating, as adjusted models and sensitivity analyses suggest potential confounding by patient risk and procedural complexity. Prospective studies with standardized complication severity grading and detailed preoperative pulmonary characterization are needed.

## 1 Introduction

Despite advances in surgical and anesthesiologic techniques, anatomical lung resections continue to carry a high risk of postoperative pulmonary complications. Complications arising from such procedures, including pneumonia, atelectasis, respiratory insufficiency and acute respiratory distress syndrome (ARDS), are particularly prevalent in cases involving one-lung ventilation (OLV) (1–4).

The approach of choice to achieving OLV involves the use of double-lumen tubes (5). While this approach engenders favorable surgical conditions, it concomitantly subjects the ventilated lung to augmented mechanical and inflammatory stress. This has given rise to concerns regarding the potential for ventilator-induced lung injury and perioperative hypoxemia (6–8).

In light of the aforementioned concerns, strategies for lung-protective ventilation (LPV) have been partly adopted in thoracic surgery (9, 10). The consistent application of these measures during thoracic surgery, particularly in the context of OLV, exhibits significant variability across medical centers and remains non-standardized (10–13).

Intraoperative ventilation parameters such as tidal volume (TV) per predicted body weight (PBW), positive end-expiratory pressure (PEEP), driving pressure (DP), and maximum airway pressure (Pmax) are now recognized as key determinants of postoperative outcomes. The optimum intraoperative settings for patients undergoing anatomical lung resection with OLV are a topic that continues to be the subject of debate (9–11, 14–17).

The present study explores the correlation between intraoperative ventilation parameters and postoperative complications in patients who have undergone anatomical lung resections with OLV. The investigation analyses data from the German Thoracic Registry (GTR). Given the retrospective registry-based design, the objective was not to establish causal ventilation thresholds, but to identify clinically relevant warning ranges associated with increased postoperative risk and to generate hypotheses for future prospective studies.

## 2 Methods

### 2.1 Study design

This study constitutes a secondary analysis of data from a larger multicenter cohort study, titled “Impact of Intraoperative Ventilation Parameters on Postoperative Outcomes in Thoracic Surgery: A Multicenter Registry-Based Analysis,” which included 2,922 patients. Within the original cohort, patients undergoing anatomical lung resections were identified as a particularly vulnerable subgroup regarding postoperative complications. The present analysis was therefore designed to specifically investigate this subgroup in greater detail.

Given the retrospective registry-based design, this analysis is subject to inherent limitations, including selection bias and incomplete documentation. Eligibility required complete recording of key intraoperative ventilation variables, which may preferentially include centers or cases with more complete charting and exclude patients with missing fields. Consequently, 1128 anatomical resections were available for univariable analyses, whereas multivariable models were restricted to complete cases (*n* = 983), and to *n* = 886 after excluding postoperative technical surgical complications in the prespecified sensitivity analysis. Missing data were not imputed; therefore, if missingness was not completely at random, effect estimates may be biased.

The study was conducted as a retrospective, multicenter cohort analysis across eight thoracic surgical centers in Germany, based on data from the GTR collected between January 2015 and December 2021. The time frame was selected because it encompassed the years during which complete, harmonized registry documentation was available for the study question. However, practice evolution over time was considered in the analysis.

Despite the fact that the registry encompasses a wide range of thoracic procedures, the present analysis concentrated on a predefined subgroup of anatomical resections with complete intraoperative ventilation documentation. This reduces the number of eligible cases per center-year.

The GTR is an interdisciplinary, multicenter benchmarking registry under the patronage of the German Society of Anesthesiology and Intensive Care Medicine (DGAI) and the German Society of Thoracic Surgery (DGT), capturing standardized perioperative quality indicators from admission to discharge via a web-based platform (18). The GTR provides a comprehensive and standardized dataset covering the full perioperative course of anatomical lung resection, including preoperative, intraoperative, and postoperative information. Participation in the registry is contingent upon the performance of a minimum of 50 thoracic procedures per year by each center.

The objective of this analysis was to examine the association between intraoperative ventilation parameters during OLV and the incidence of postoperative complications, respiratory complications, and in-hospital mortality in patients undergoing anatomical lung resection.

### 2.2 Patient selection

The study comprised adult patients who underwent anatomical lung resection with intraoperative OLV and had complete documentation of key ventilation parameters. Inclusion criteria demanded an OLV duration greater than zero minutes, the utilization of a documented controlled ventilation mode – either pressure-controlled ventilation (PCV) or volume-controlled ventilation (VCV) – and valid entries for PEEP, Pmax, respiratory rate, TV/PBW, and minute volume. Furthermore, documented body weight and height were necessary for the calculation of PBW.

Patients were excluded from the study if they received spontaneous or jet ventilation, or if essential documentation fields were missing. Furthermore, a prespecified sensitivity analysis excluded patients with postoperative procedure-related surgical complications, including bronchial stump insufficiency and/or persistent fistula or air leak for more than seven days. The objective of this analysis was to evaluate the robustness of observed associations between ventilation parameters and outcomes after excluding events potentially dominated by procedure-related, parenchymal, or case-complexity factors rather than by ventilation-related lung injury alone. These events were not interpreted as defects in surgical technique, since prolonged air leak may also occur in patients with emphysema, COPD, impaired lung parenchyma, or complex resections. Nevertheless, it must be noted that this approach may give rise to selection (or collider) bias if technical complications are found to be associated with both case complexity and postoperative pulmonary outcomes.

Intraoperative technical complications were not recorded consistently within the registry and therefore could not be reliably excluded.

### 2.3 Ventilation and surgical parameters

The intraoperative ventilation parameters that were extracted from the dataset included the DP, calculated as the difference between the Pmax and PEEP; PEEP; Pmax; the TV/PBW; and the ventilation mode, either PCV or VCV. The surgical parameters that were included in the study encompassed the type of anatomical resection (e.g., lobectomy or segmentectomy), the surgical approach (video-assisted thoracoscopic surgery [VATS] versus thoracotomy), the duration of surgery, and the duration of OLV. Furthermore, the airway management techniques employed were meticulously documented, with particular attention paid to the utilization of either a double-lumen tube or a bronchial blocker.

### 2.4 Outcome parameters

The primary outcomes were defined as the occurrence of postoperative complications, characterized by any deviation from the expected clinical course that necessitated medical intervention. The composite endpoint of respiratory complications was assessed, incorporating a range of conditions including pneumonia, respiratory insufficiency, acute respiratory distress syndrome (ARDS), re-intubation, non-invasive ventilation (NIV), extracorporeal membrane oxygenation (ECMO), and unplanned admission to the intensive care unit (ICU). Surgical technical complications (e.g., bronchial stump insufficiency/prolonged air leak) were not included in this respiratory composite endpoint. Severity grading according to the Ottawa Thoracic Morbidity and Mortality classification was not available in the registry extract used for this analysis. A retrospective OTMM assignment was not performed because the registry did not consistently capture the required details on severity grade, intervention level, organ-system attribution, and clinical course. Therefore, the present analysis reports predefined complication categories and in-hospital mortality rather than OTMM severity grades.

The secondary outcome was in-hospital mortality, defined as death from any cause during the index hospital stay*.*

### 2.5 Data collection

The data were prospectively recorded in the GTR using a standardized, pseudonymized electronic data sheet and subsequently extracted for statistical analysis. All surgical procedures were performed in accordance with the institution's guidelines for quality assurance in perioperative medicine. All outcomes were derived from predefined postoperative complication fields of the GTR. Complications were documented at each participating center by local clinical staff based on routine clinical documentation using the registry's standard definitions.

The following preoperative variables were taken into consideration: age, sex, body mass index (BMI), American Society of Anesthesiologists (ASA) status, smoking history, comorbidities, and laboratory parameters. The intraoperative data comprised ventilation settings, the duration of the procedure, and the methods employed in airway management. Postoperative outcomes were documented until hospital discharge or in-hospital death.

The registry extract used for this analysis did not contain sufficiently complete structured variables on chronic bronchopulmonary pathology, including COPD and emphysema, or preoperative pulmonary function parameters such as FEV1, FVC, and DLCO. These variables could therefore not be included as covariates, used for subgroup analyses, or assessed with regard to anesthesia duration, OLV duration, or postoperative complications. Available proxies for baseline pulmonary risk included smoking status, preoperative respiratory infection, ASA status, BMI, and the recorded comorbidity fields.

### 2.6 Statistical analysis

The calculation of descriptive statistics was conducted for both baseline and procedural variables. Categorical variables are reported as absolute and relative frequencies and were compared using chi-squared (*χ*^2^) tests. The continuous variables were then compared using t-tests or Mann–Whitney U tests, depending on the data distribution.

An exploratory threshold analysis was performed, guided by existing literature and clinical relevance (DP >15, >20, >25 mbar; PEEP >7, >8 mbar; Pmax >25, >30 mbar; TV >5 mL/kg PBW). In order to estimate independent associations with outcomes, multivariable modified Poisson regression with robust variance estimation was used to obtain adjusted relative risks (aRR) and Wald tests for the primary outcomes in the anatomical resection cohort (*n* = 983). The models were adjusted for age, BMI, sex (female vs male), year of surgery, surgical duration, OLV time, DP, mean PEEP, TV/PBW, mean FiO₂, mean Pmax, surgical approach, ASA group, and center. The ventilation mode was entered using dummy variables with PCV designated as the reference category. A prespecified sensitivity analysis was conducted, which excluded patients with postoperative technical surgical complications (persistent bronchial stump insufficiency and/or fistula for >7 days), yielding *n* = 886. As intraoperative technical complications were not systematically recorded in the registry, they could not be excluded. Instead, they were accounted for indirectly by adjustment for surgical duration. All statistical tests were two-tailed, with a significance level set at *p* < .05. Statistical analyses were performed using SPSS Version 29.0.2.0.

No additional regression models including COPD, emphysema, FEV1, FVC, DLCO, or OTMM severity grades were performed, because these variables were not available in a sufficiently complete and standardized format in the registry extract.

### 2.7 Ethical considerations

Approval for the study was granted by the institutional review board of the University of Witten/Herdecke (approval no. 64-2014). The General Data Protection Regulation guidelines were followed in the pseudonymization of all data. Prior to enrolment in the GTR, written informed consent was obtained from all patients.

## 3 Results

### 3.1 Demographic data

The analysis included a total of 1128 patients who underwent anatomical lung resections. Most patients were male (63.3%), while 36.7% were female. Regarding smoking status, 30.5% were current smokers, 33.6% were former smokers (quit >3 months before surgery), and 35.9% had never smoked. A preoperative respiratory infection within four weeks was present in 5.9% of patients.

Concerning comorbidities, 14.0% had diabetes mellitus, 10.6% had renal insufficiency, 15.7% suffered from Coronary arterial disease (CAD), and 6.5% had peripheral arterial disease (PAD). A previous stroke or transient ischemic attack (TIA) was reported in 6.0% of the cohort. Obstructive sleep apnea syndrome (OSAS) was noted in 4.8%, and chronic pain syndromes in 5.0% of patients.

Regarding preoperative oncologic treatment, 79.5% had received no prior chemotherapy, radiotherapy, or lung surgery. However, 13.7% had undergone chemotherapy, 6.0% received radiotherapy, and 14.3% had a prior lung surgery.

Detailed chronic bronchopulmonary diagnoses, including COPD and emphysema, as well as preoperative pulmonary function parameters such as FEV1, FVC, and DLCO, were not available in a sufficiently complete structured format in the analyzed registry extract. Therefore, their distribution and their association with anesthesia duration, OLV duration, and postoperative complications could not be reported.

### 3.2 Complications

**Figure 1:**
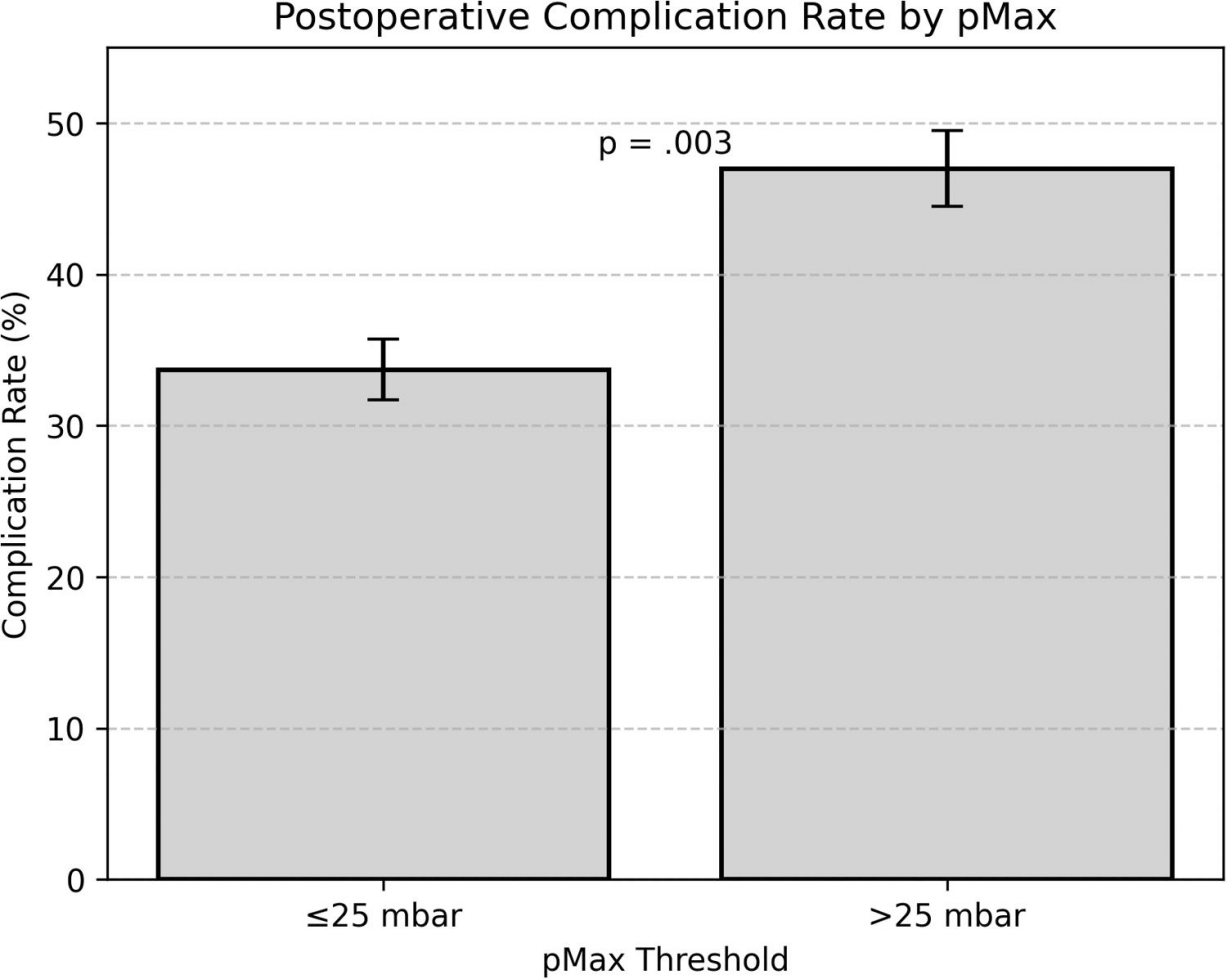
Postoperative complication rates by intraoperative peak airway pressure (Pmax) in anatomical lung resections. Complications increased from 33.7% (≤25 mbar) to 47.0% (>25 mbar) (*p* = .003).

Postoperative complications occurred in 28.7% (*n* = 324) of patients. From a clinical perspective, elevated airway pressure and volume thresholds have been demonstrated to be significantly associated with a heightened prevalence of complications. Specifically, DP levels in excess of 20 mbar were found to be associated with an increase in complication rates from 34.1% to 47.5% (*p* = .008). In a similar manner, Pmax >25 mbar was associated with an increase from 33.7% to 47.0% (*p* = .003; Figure 1), and Pmax >30 mbar with an increase from 34.8% to 59.1% (*p* = .018). Furthermore, the administration of a dose of 5 mL/kg PBW via the TV was associated with an increase in complication rates from 28.8% to 37.2% (*p* = .013). The detailed results are provided in Table 1. The findings of this study indicate that exceeding commonly utilized airway pressure and tidal volume limits during OLV in anatomical resections is associated with a clinically significant increase in postoperative morbidity.

**Table 1:** Results of complications in the subgroup anatomical resection.

Parameter	Comparison value (%)	Complication rate (%)	*χ*^2^ (df = 1)	*p*-value
DP > 15 mbar	34.3	37.2	0.96	.328
DP > 20 mbar	34.1	47.5	7.06	.008
Pmax > 20 mbar	33.5	37.6	2.01	.157
Pmax > 25 mbar	33.7	47.0	8.94	.003
Pmax > 30 mbar	34.8	59.1	5.57	.018
PEEP > 8 mbar	34.7	43.8	2.50	.114
PEEP > 7 mbar	34.3	40.7	2.61	.107
TV > 5 mL/kg PBW	28.8	37.2	6.14	.013

DP, driving pressure; Pmax, maximum airway pressure; PEEP, positive end-expiratory pressure; TV, tidal volume; df, degrees of freedom.

For the primary outcome of the regression model, overall complications, the year of surgery was inversely associated with complications (*p* < .001). A longer duration of surgery was found to be associated with an elevated risk of complications (*p* = .032). Furthermore, higher DP was observed to be associated with a moderately increased risk (*p* = .042). The female sex was found to be associated with a reduced risk of complications (*p* = .023). In the sensitivity analysis excluding technical complications, the inverse association with year of surgery persisted (*p* < .001), whereas the associations with driving pressure (*p* = .163) and surgical duration (*p* = .077) were attenuated. The ventilation mode (VCV vs PCV) was not associated with complications in either analysis (full cohort: *p* = .909; sensitivity: *p* = .945). Full results are displayed in Table 2.

**Table 2:** Multivariable modified Poisson regression (robust variance) for overall complications in anatomical resections, reported as aRR (95% CI) with Wald *p*-values; full cohort and sensitivity analysis excluding technical complications.

Predictor (unit)	RR (model)	95% CI	*p* (Wald)	RR (sensitivity)	95% CI	*p* (Wald)
Age (per year)	1.002	0.993–1.010	.698	1.000	0.990–1.010	.975
BMI (per kg/m^2^)	0.987	0.968–1.006	.182	1.001	0.977–1.025	.965
Year of surgery	0.811	0.738–0.892	<.001	0.822	0.732–0.924	<.001
Surgery time	1.004	1.000–1.007	.032	1.004	1.000–1.008	.077
OLV (per min)	0.998	0.995–1.002	.329	0.998	0.994–1.003	.432
DP(per mbar)	1.024	1.001–1.047	.042	1.023	0.991–1.056	.163
PEEP (per mbar)	0.994	0.944–1.046	.808	0.983	0.919–1.052	.624
TV/PBW (per kg)	0.995	0.966–1.025	.736	0.984	0.932–1.039	.570
FiO₂ (mean)	1.183	0.516–2.713	.692	1.717	0.603–4.888	.312
Female vs Male	0.807	0.670–0.971	.023	0.849	0.677–1.064	.154
VCV vs PCV	0.985	0.767–1.266	.909	1.013	0.705–1.455	.945

aRR, adjusted relative risk; CI, confidence interval; OLV, one-lung ventilation; PEEP, positive end-expiratory pressure; FiO₂, fraction of inspired oxygen; Pmax, maximum airway pressure; TV/PBW; tidal volume per predicted body weight; ASA, American Society of Anesthesiologists physical status.

### 3.3 Respiratory complications

A total of 16.3% (*n* = 184) experienced respiratory complications, which included respiratory insufficiency, pneumonia, ARDS, non-invasive ventilation (NIV), reintubation, ECMO, or unplanned ICU admission. Surgical technical complications (e.g., bronchial stump insufficiency/bronchopleural fistula or prolonged air leak) were analyzed separately and excluded in sensitivity analyses. These univariable patterns provide a pragmatic clinical interpretation of how frequently respiratory events occurred when common intraoperative “warning thresholds” for airway pressures and PEEP were exceeded.

A rise in DP above 15 mbar was found to be associated with a significant increase in complication rates from 14.2% to 19.6% (*p* = .026). A further increase in DP above 20 mbar resulted in complication rates rising from 15.4% to 25.6% (*p* = .006), while a threshold of 25 mbar was linked to a rise from 15.9% to 31.4% (*p* = .006).

With regard to Pmax, a significant increase in complication rates was observed when Pmax exceeded 20 mbar, rising from 13.9% to 20.8% (*p* = .015). Furthermore, Pmax values greater than 25 mbar were found to be associated with an increase from 14.7% to 25.2% (*p* = .001). When Pmax exceeded 30 mbar, the complication rate exhibited a significant increase from 16.0% to 33.3% (*p* = .005).

**Figure 2:**
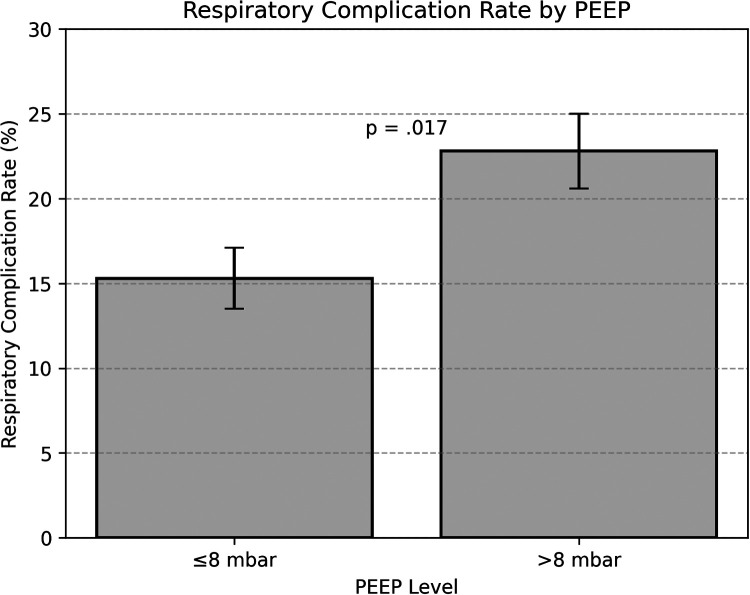
Respiratory complication rates by intraoperative positive end-expiratory pressure (PEEP) level. A PEEP >8 mbar was associated with a significantly higher rate of respiratory complications (22.8%) compared to ≤8 mbar (15.3%, *p* = .017).

PEEP was also found to have a significant impact on the outcomes. A PEEP level more than 8 mbar was found to result in an increased complication rate from 15.3% to 22.8% (*p* = .017), this is shown in Figure 2. In a similar manner, a PEEP threshold of 7 mbar was found to be associated with an increase from 15.0% to 20.8% (*p* = .033).

The complete results have been collated and are displayed in Table 3. The risk of respiratory complications increased substantially with longer durations of OLV and surgery. Patients with OLV durations >180 min had a 30.3% complication rate, compared to 5.9% at ≤30 min (*χ*^2^(6) = 90.34, *p* < .001). Similar trends were observed for prolonged surgery times (*χ*^2^(6) = 101.52, *p* < .001). In this cohort, there was a marked increase in respiratory morbidity once airway pressures exceeded the reported thresholds. This supports the hypothesis that a lung-protective focus should be adopted during OLV in anatomical resections.

**Table 3:** Results of respiratory complications in subgroup anatomical resection.

Parameter	Comparison value (%)	Complication rate (%)	*χ*^2^ (df = 1)	*p*-value
DP > 15 mbar	14.2	19.6	4.97	.026
DP > 20 mbar	15.4	25.6	7.52	.006
DP > 25 mbar	15.9	31.4	7.65	.006
Pmax > 20 mbar	13.9	20.8	5.91	.015
Pmax > 25 mbar	14.7	25.2	10.87	.001
Pmax > 30 mbar	16.0	33.3	8.05	.005
PEEP > 8 mbar	15.3	22.8	5.69	.017
PEEP > 7 mbar	15.0	20.8	4.55	.033

DP, driving pressure; Pmax, maximum airway pressure; PEEP, positive end-expiratory pressure; TV, tidal volume; df, degrees of freedom.

In our regression model for the composite outcome of respiratory complications, the year of surgery was found to be inversely associated with respiratory events (full cohort: *p* < .001; sensitivity: *p* = .025). A positive correlation was identified between the duration of surgery and the risk of respiratory complications (full cohort: *p* = .020; sensitivity: *p* = .031). The female sex was found to be strongly associated with a reduced risk of lower respiratory complications (full cohort: *p* < .001; sensitivity: *p* = .007). In the full cohort, BMI demonstrated an inverse association with respiratory complications (*p* < .001). However, this association was not significant in the sensitivity analysis (*p* = .250). Importantly, in multivariable models adjusting for patient risk, procedural duration, temporal trends, and center effects, ventilation parameters (driving pressure, mean PEEP, TV/PBW, mean FiO2, and ventilation mode) were not independently associated with respiratory complications, suggesting that the univariable threshold associations may partly reflect underlying patient risk and procedural complexity rather than a direct ventilation effect. Full results are shown in Table 4.

**Table 4:** Multivariable modified Poisson regression (robust variance) for respiratory complications in anatomical resections, reported as aRR (95% CI) with Wald *p*-values; full cohort and sensitivity analysis excluding technical complications.

Predictor (unit)	RR (model)	95% CI	*p* (Wald)	RR (sensitivity)	95% CI	*p* (Wald)
Age (per year)	1.004	0.992–1.017	.490	1.000	0.983–1.018	.986
BMI (per kg/m^2^)	0.951	0.925–0.978	<.001	0.977	0.938–1.017	.250
Year of surgery	0.778	0.686–0.883	<.001	0.813	0.677–0.975	.025
Surgery time	1.005	1.001–1.010	.020	1.006	1.001–1.011	.031
OLV (per min)	0.998	0.993–1.002	.269	0.997	0.992–1.003	.374
DP (per mbar)	1.020	0.986–1.056	.247	0.990	0.927–1.057	.759
PEEP (per mbar)	1.034	0.961–1.111	.371	0.960	0.883–1.044	.340
TV/PBW (per kg)	0.997	0.961–1.033	.854	1.030	0.924–1.148	.594
FiO₂ (mean)	1.163	0.349–3.876	.806	3.685	0.573–23.685	.169
Female vs Male	0.596	0.452–0.785	<.001	0.562	0.370–0.853	.007
VCV vs PCV	0.983	0.699–1.383	.922	0.972	0.504–1.874	.932

aRR, adjusted relative risk; CI, confidence interval; OLV, one-lung ventilation; PEEP, positive end-expiratory pressure; FiO₂, fraction of inspired oxygen; Pmax, maximum airway pressure; TV/PBW, tidal volume per predicted body weight; ASA, American Society of Anesthesiologists physical status.

### 3.4 Mortality

In this group 1.2% (*n* = 14) died during their hospital stay. In the unadjusted analysis of mortality of these patients undergoing anatomical resection, no statistically significant associations were found between the evaluated ventilation parameters and mortality rates. Only increased durations of both OLV and surgery were significantly associated with in hospital mortality (*p* < .001 for both). Table 5 shows the full results of this analysis.

**Table 5:** Results of mortality subgroup anatomical resection.

Parameter	Comparison value (%)	Complication rate (%)	*χ*^2^ (df = 1)	*p*-value
DP > 20 mbar	1.2	2.0	0.54	.464
Pmax > 20 mbar	1.3	1.2	0.01	.922
Pmax > 25 mbar	1.1	2.3	1.30	.255
Pmax > 30 mbar	1.3	0.0	0.28	.595
PEEP > 8 mbar	1.2	1.4	0.01	.918
PEEP > 7 mbar	1.4	0.6	0.73	.396

DP, driving pressure; Pmax, maximum airway pressure; PEEP, positive end-expiratory pressure; df, degrees of freedom.

In exploratory multivariable modified Poisson regression models, mortality analyses showed numerical instability (singular Hessian) due to the low number of events; estimates should therefore be interpreted cautiously. In the full cohort, higher mean PEEP was associated with lower mortality (aRR 0.619 per mbar, 95% CI 0.451–0.849; *p* = .003), and this association persisted in the sensitivity analysis excluding postoperative surgical technical complications (aRR 0.585 per mbar, 95% CI 0.429–0.798; *p* < .001). No other covariates reached statistical significance, including year of surgery (full cohort: *p* = .351; sensitivity: *p* = .858). Full results are shown in Table 6. Overall, univariable threshold analyses consistently demonstrated higher postoperative and respiratory complication rates when airway pressures and TV exceeded commonly used lung-protective ranges. Conversely, multivariable models indicated that only specific parameters (and not ventilation mode) remained independently associated after adjustment. Mortality was rare, and adjusted mortality estimates should therefore be interpreted with caution as exploratory.

**Table 6:** Multivariable modified Poisson regression for in-hospital mortality in anatomical resections shown as aRR with 95% CI and Wald *p*-values; full cohort and sensitivity analysis excluding technical complications.

Predictor (unit)	RR (model)	95% CI	*p* (Wald)	RR (sensitivity)	95% CI	*p* (Wald)
Age (per year)	1.048	0.989–1.112	.115	1.030	0.970–1.095	.334
BMI (per kg/m^2^)	0.946	0.828–1.081	.413	0.982	0.866–1.114	.782
Year of surgery	1.537	0.623–3.790	.351	0.912	0.332–2.502	.858
Surgery time	1.000	0.978–1.022	.975	0.992	0.977–1.008	.352
OLV time (per min)	1.010	0.985–1.035	.433	1.020	0.999–1.040	.059
DP (per mbar)	0.920	0.744–1.136	.438	0.854	0.672–1.085	.197
PEEP (per mbar)	0.619	0.451–0.849	.003	0.585	0.429–0.798	<.001
TV/PBW (per kg)	1.015	0.972–1.060	.495	1.020	0.984–1.057	.281
FiO₂ (mean)	383.760	0.383–384582.066	.091	310.948	0.117–826254.523	.154
Female vs Male	1.128	0.296–4.301	.860	1.360	0.333–5.566	.669

aRR, adjusted relative risk; CI, confidence interval; OLV, one-lung ventilation; PEEP, positive end-expiratory pressure; FiO₂, fraction of inspired oxygen; Pmax, maximum airway pressure; TV/PBW, tidal volume per predicted body weight; ASA, American Society of Anesthesiologists physical status.

## 4 Discussion

In this multicenter, registry-based analysis of 1128 patients undergoing anatomical lung resection with OLV, elevated intraoperative airway pressures and TVs were observed to be significantly associated with an increased risk of postoperative and respiratory complications. Specifically, a DP exceeding 20 mbar, a Pmax >25 mbar, a TV >5 mL/kg PBW and PEEP > 8mbar emerged as relevant thresholds. In-hospital mortality was low (1.2%), limiting statistical power and the stability of multivariable modelling. Accordingly, associations with ventilation parameters should be interpreted as exploratory; in adjusted models, higher mean PEEP was inversely associated with mortality, while unadjusted analyses suggested higher mortality with prolonged OLV and longer surgery duration. The prespecified sensitivity analysis, excluding postoperative technical surgical complications, provides additional context for interpretation. In this restricted cohort, the associations of driving pressure and surgical duration with overall complications were attenuated compared with the complete-case model. This suggests that part of the unadjusted and adjusted signal may reflect procedural complexity or technical factors that are imperfectly captured in registry data. It is important to note that intraoperative technical complications were not systematically recorded and could not be excluded. Furthermore, exclusion based on postoperative events may induce selection mechanisms. Therefore, the sensitivity analysis should be interpreted as a robustness check rather than a definitive separation of “ventilation-caused” versus “surgery-caused” complications.

The findings of this study are consistent with those of previous studies that identified DP as a robust predictor of pulmonary injury in both intensive care and perioperative settings (9, 19–21). In accordance with the findings of Park et al., who reported a higher incidence of pulmonary complications with elevated DP during OLV in thoracic surgery (19), our study demonstrates a significant increase in both general (*p* = .008) and respiratory complications (*p* = .006) when DP exceeded 20 mbar.

The observed increase in postoperative and respiratory morbidity at higher intraoperative airway pressures and tidal volumes is biologically plausible in the setting of one-lung ventilation (6, 22). During OLV, the ventilated lung is substantially reduced, such that a given tidal volume may translate into higher regional strain and overdistension (6). Elevated DP, when considered as an integrative surrogate of respiratory system compliance, has been demonstrated to reflect higher stress applied to the ventilated lung. This has been linked to ventilator-induced lung injury pathways (9, 19). High peak airway pressures have been demonstrated to contribute to barotrauma and microvascular injury, while repeated opening and closing of unstable lung units (atelectrauma) has been shown to amplify local inflammation (22). Such insults may trigger a systemic inflammatory response, known as “biotrauma”, which can potentially contribute to pneumonia, respiratory insufficiency, and other postoperative pulmonary complications (23).

The significant correlation of Pmax >25 mbar and TV >5 mL/kg PBW and postoperative complications provides further support for the growing body of evidence in favor of lung-protective intraoperative ventilation, despite ongoing debate about it (6, 7, 24, 25).

High peak pressures have been demonstrated to potentially contribute to barotrauma and local inflammatory responses, particularly in the ventilated lung during OLV, while other findings in the intensive care unit shows moderate PEEP is suggested to be protective (11, 17, 26–29). While the threshold of TV >5 mL/kg PBW is in line with the traditional intraoperative standards, it aligns with LPV principles derived from ARDS management (30). The findings of this study are consistent with the current body of evidence and serve to reinforce the importance of the implementation of lung-protective ventilation thresholds and strategies during thoracic surgery and anatomical lung resections.

It was found that elevated PEEP >8 mbar was significantly associated with respiratory complications (*p* = .017). Nevertheless, the exact role of PEEP in the literature remains to be elucidated. While individualized PEEP has been shown to prevent atelectasis, improve oxygenation and may be protective (16, 28, 31), excessive levels have the potential to impair hemodynamics and contribute to overdistension, particularly in reduced lung volumes during OLV (27, 32). These findings underscore the necessity for individualized PEEP titration and imply that both insufficient and excessive levels may prove deleterious.

The association between higher PEEP and respiratory complications in univariable analyses should be interpreted cautiously. While adequate PEEP can prevent derecruitment and reduce atelectrauma, excessive PEEP in the context of reduced ventilated lung volume during OLV may promote overdistension, impair right ventricular afterload, and compromise hemodynamics (33, 34). Taken together, these considerations support an individualized approach to PEEP titration rather than uniform “high” or “low” settings (33).

Furthermore, the strong associations observed for prolonged durations of OLV and surgery align with previous data indicating that increased surgical complexity and exposure time can exacerbate the risk of systemic inflammation, ventilator-induced lung injury and postoperative complications (6, 33). Concurrently, in-hospital mortality was minimal in this cohort (1.2%, *n* = 14), thereby diminishing the statistical capacity to discern ventilation-related mortality effects. This finding endorses the prioritization of clinically significant postoperative pulmonary complications as primary outcomes in forthcoming studies.

The threshold-based associations observed in unadjusted analyses should be interpreted in the context of potential confounding by case complexity and “confounding by indication”. Higher airway pressures or PEEP may be applied in response to worse compliance, more advanced lung disease, challenging surgical conditions, or prolonged OLV, all of which independently increase postoperative risk. Registry data is unable to fully capture time-varying ventilator adjustments, recruitment maneuvers, or transpulmonary pressures; therefore, residual confounding cannot be excluded. From a clinical perspective, the identified thresholds may still serve as pragmatic warning markers, prompting a reassessment of ventilator settings and lung mechanics.

Because in-hospital mortality was rare, the mortality models should be regarded as exploratory and potentially unstable. The observed association of mortality with prolonged OLV and longer surgery duration in unadjusted analyses likely reflects underlying procedural complexity and patient risk. An inverse association between mean PEEP and mortality was observed in adjusted models; however, given model instability and residual confounding, this finding should be interpreted cautiously and validated in prospective studies. However the observation of the unadjusted analysis aligns with previous data indicating that increased surgical complexity and exposure time can exacerbate the risk of systemic inflammation, ventilator-induced lung injury and postoperative complications, but the effect on mortality remains a subject of discussion (14, 35, 36). Although ventilation settings can be modified during surgery, case duration often reflects underlying patient and procedural complexity, which may be more difficult to optimize.

The present findings are consistent with the results of randomized and observational studies, which demonstrate that lung-protective strategies in thoracic surgery may result in improved postoperative outcomes (11–13, 16, 24). However, variability in intraoperative practices across centers, the lack of international clinical practice guideline on the protective lung ventilation strategy for thoracic anesthesia - as reported by Kuo et al. (10)—continues to hinder standardization. The present study contributes by providing empirically derived threshold values from a large national registry, which may inform the development of guidelines and clinical decision-making.

This multicenter study provides a large registry-based analysis, focusing exclusively on the intraoperative ventilation parameters of anatomical lung resections under OLV as recorded in the database. The utilization of a standardized national dataset amassed over a period of seven years serves to enhance the robustness and external validity of the findings, as it reflects real-world clinical practice across a range of thoracic surgical centers. The analysis's ability to avoid heterogeneity, which is commonly introduced by the pooling of different thoracic procedures, is due to the isolation of a well-defined surgical subgroup. This, in turn, allows for more specific conclusions regarding ventilation strategies in anatomical resections.

However, it is important to acknowledge several limitations. Firstly, the retrospective design precludes causal inference and allows for residual confounding despite multivariable adjustment. Secondly, selection bias is probable due to the necessity of comprehensive documentation of key ventilation variables, with regression models being based on complete cases. Consequently, excluded patients may exhibit systematic differences with respect to baseline risk, procedural complexity, or documentation quality. Thirdly, as with any registry-based analysis, data quality may be affected by missing entries, inter-center variability in documentation, and misclassification or underreporting of complications, which could result in a bias towards either direction in effect estimates. Fourthly, several potentially relevant intraoperative and perioperative factors were not captured consistently (e.g., recruitment maneuvers, real-time ventilator adjustments, postoperative respiratory interventions), and intraoperative technical complications were not recorded in a standardized manner. Thus, confounding by procedural complexity cannot be fully excluded, even though surgical duration was included as a proxy. In addition, the registry extract did not provide sufficiently complete structured data on chronic bronchopulmonary disease, including COPD and emphysema, or on preoperative lung function parameters such as FEV1, FVC, and DLCO. We therefore could not assess whether impaired baseline pulmonary function influenced ventilation parameters, anesthesia or OLV duration, postoperative complications, or prolonged air leak. This represents an important limitation, because chronic parenchymal lung disease may affect both intraoperative respiratory mechanics and postoperative pulmonary outcomes. Furthermore, complications were not graded according to the Ottawa Thoracic Morbidity and Mortality classification in the registry. Retrospective OTMM grading was not considered valid because the necessary information on severity, intervention level, organ-system attribution, and clinical course was not consistently available. Therefore, this study cannot provide a severity-stratified analysis of postoperative morbidity. Non-respiratory reasons for ICU admission or other neurological, vascular, or systemic complications may also have contributed to the composite endpoints and could not be fully separated. Fifthly, it is important to note that variation in center-specific ventilation practices, which reflect routine care, may also have introduced systematic differences that are difficult to fully account for. Finally, formal multicollinearity diagnostics were not performed, despite the conceptual correlation between ventilation variables (DP, Pmax, TV). This may have reduced the precision of the adjusted estimates, and the multivariable results should be interpreted with caution. In conclusion, the findings were confined to in-hospital occurrences, and the modest mortality rate imposed limitations on the statistical power and model stability for mortality analyses.

In addition the study period (2015–2021) witnessed significant developments in perioperative thoracic care, notably the publication of ERAS® Society/ESTS guidelines for enhanced recovery after lung surgery (2019), which reinforced recommendations for lung-protective intraoperative strategies and impacted routine practice across numerous centers (37). Consequently, calendar-time effects and evolving perioperative pathways (e.g., rehabilitation and minimally invasive approaches) may confound observed associations. Although we adjusted for calendar year, residual confounding by secular trends and evolving perioperative pathways may persist; future work could consider stratified analyses (e.g., pre- vs. post-2019 eras) to further assess robustness.

In order to further advance current knowledge in this field, future research should concentrate on the conducting of prospective, randomized controlled trials. These are essential to confirm the associations identified in observational data and to determine whether intraoperative ventilation strategies have a direct causal impact on postoperative outcomes. To enhance the comprehension of the mechanisms underpinning ventilator-associated lung injury, experimental studies incorporating parameters such as dynamic compliance, inflammatory markers, and real-time diagnostics — including lung ultrasound or advanced CT-based aeration assessments — are to be undertaken. Concurrently, the implementation of adaptive ventilation approaches, incorporating AI-supported decision systems, holds promise for enhancing intraoperative respiratory management.

Prospective research directions. It is recommended that future studies extend beyond generic comparisons of ventilation modes and encompass the testing of physiology-driven strategies. Pragmatic multicenter trials could randomize patients undergoing anatomical lung resection with OLV to either a DP –guided ventilation algorithm (targeting low DP by adjusting TV, PEEP, and recruitment maneuvers) versus usual care, and/or to individualized PEEP titration (e.g., best-compliance, EIT-guided, or transpulmonary pressure–guided) versus fixed PEEP. In view of the low in-hospital mortality observed, primary endpoints should priorities clinically meaningful postoperative pulmonary complications and respiratory composite outcomes, complemented by mechanistic endpoints such as dynamic compliance trajectories and perioperative inflammatory biomarkers. The cluster or stepped-wedge implementation design may be particularly suitable for the evaluation of protocolized lung-protective ventilation and its integration into future guideline recommendations. Furthermore, long-term observational studies should examine the effects of intraoperative lung-protective strategies on functional recovery, pulmonary capacity, and overall quality of life after thoracic surgery.

## 5 Conclusion

Intraoperative DP (Pmax–PEEP) levels in excess of 20 mbar, Pmax levels exceeding 25 mbar, tidal volumes in excess of 5 mL/kg PBW, and PEEP levels in excess of 8 mbar were found to be significantly associated with higher complication rates in anatomical lung resections under one-lung ventilation.

Clinically, these thresholds, which are easily monitorable, should be used as pragmatic intraoperative alert points. These should prompt a structured reassessment of lung mechanics and ventilator settings (e.g., reduction of tidal volume and airway pressures, and individualized PEEP titration with close hemodynamic monitoring) rather than as strict causal cut-offs.

These findings provide a compelling rationale for the broader implementation and standardization of lung-protective ventilation protocols in thoracic anesthesia. Furthermore, they offer a robust foundation for prospective physiology-guided studies, which may serve to validate threshold-based algorithms and inform future recommendations.

## Data Availability

The datasets presented in this article are not readily available because they are the intellectual property of the Akademie der Unfallchirurgie (AUC) GmbH as part of the TraumaRegister DGU®, and access to them is strictly controlled. Only upon request to the registry holder and subject to approval, data use agreements, and applicable data protection regulations, can access be granted. Consequently, the authors are not permitted to share the raw data directly. Requests to access these datasets should be directed to mark.gerbershagen@uni-wh.de.
